# Effect of Interfacial Interaction on the Demolding Deformation of Injection Molded Microfluidic Chips

**DOI:** 10.3390/nano12193416

**Published:** 2022-09-29

**Authors:** Yilei Wang, Can Weng, Huijie Sun, Zijian Deng, Bingyan Jiang

**Affiliations:** 1 College of Mechanical and Electrical Engineering, Central South University, Changsha 410083, China; 2 State Key Laboratory of High Performance Complex Manufacturing, Central South University, Changsha 410083, China

**Keywords:** microfluidic chip, injection molding, interfacial interaction, demolding deformation, molecular dynamics

## Abstract

During the demolding process, the interfacial interaction between the polymer and the metal mold insert will lead to the deformation of the micro-structure, which will directly affect the molding quality and performance of injection molded microfluidic chips. In this study, the demolding quality of micro-channels and micro-mixing structures of polycarbonate (PC), polymethyl methacrylate (PMMA), cyclic olefin copolymer (COC), and polystyrene (PS) microfluidic chips for heavy metal detection were investigated by molding experiments. The experimental results showed that the structures of microfluidic chips could be completely replicated. However, tensile deformation and fracture defects were observed at the edges of the micro-structures after demolding. Compared to the Ni mold insert, the calculation of the relative deviation percentages showed that the width of the micro-channel became larger and the depth became smaller, while the dimensions of the micro-mixing structure changes in the opposite direction. Subsequently, a molecular dynamics (MD) simulation model of polymer/nickel (Ni) mold insert for injection molding was established. The changes of adhesion work, demolding resistance and potential energy during demolding were analyzed. The simulation results showed that the polymer structures had some deformations such as necking, molecular chain stretching and voids under the action of adhesion work and demolding resistance. The difference in the contact area with the mold insert directly brought different interfacial interactions. In addition, the potential energy change of the polymer system could be used to quantitatively characterize the demolding deformation of the structure. Overall, the MD method is able to effectively explain the internal mechanisms of interfacial interactions, leading to the demolding deformation of polymer structures from the molecular/atomic scale.

## 1. Introduction

Microfluidic chips have been widely used in biochemical analysis [1], medical detection [2], heavy metal detection [3] and other fields, due to their miniaturization, integration and low consumption. The micro-channel network of microfluidic chips is the key structure to achieve its function, thus the molding quality of this micro-structure is particularly important [4]. Injection molding has become one of the main methods for the fabrication of polymeric micro/nano-structures, due to its high efficiency, low cost, and mass production [5]. This process consists of four stages: filling, packing, cooling and demolding.

As a critical stage that determines the quality of injection molded polymeric micro/nano-structures, demolding has been the focus of research [6,7]. When the structure is scaled down to the micro/nano-meter size, the surface and interfacial interactions between the polymer and the mold insert are significantly enhanced. As a result, some demolding deformations such as burrs, fractures and scratches can occur [8], which will directly affect the performance of the microfluidic chips [9]. In order to reduce the interfacial interactions and improve the molding quality, researchers have carried out some exploratory works in mold design [10], processing parameters optimization [4] and mold insert modification [11]. Lutey et al. [12] fabricated laser-textured micro-injection molds with different roughness and found that LIPSS oriented parallel to the injection direction achieved the greatest replication. Masato et al. [13] showed that the higher the surface roughness of the mold insert, the smaller the mechanical interlocking effect between the polymer and the mold insert. Our previous study [14] showed that increasing the mold temperature, melt temperature, and injection rate could improve the replication accuracy of the micro-channels. Modification of the mold insert surface can significantly reduce the surface roughness and surface energy, thus improving the reproduction quality of micro/nano-structures. Christine et al. [15] found that the adhesion and static friction coefficient of mold inserts modified with an organosilane coating were reduced by 50%. Li et al. [16] showed that low surface energy coatings could effectively reduce the adhesion force and the total demolding force during the hot embossing. Alborz et al. [17] concluded that coating treatments mainly reduced surface adhesion rather than improved friction properties.

However, macroscopic-scale methods of study do not provide an intuitive explanation of the intrinsic mechanisms affecting the demolding deformations of polymers. Molecular dynamics (MD) methods have been proven to be an effective method for studying molecular/atomic interfacial interactions and are widely used for different molecular systems, such as polymer–filler interfaces, polymer diffusion and tensile simulations [18,19]. Deng et al. [20] revealed the effect of functional groups on the interfacial debonding behavior through tensile experiments at the molecular-scale. Wang et al. [21] used MD simulations to conclude that non-covalently modified carbon nanotubes could further improve the ductility of cement/carbon nanotube composites. Wu et al. [22] studied the effect of mold geometry and taper on polymer deformation by using MD simulations to analyze the stress and molecular motion trajectory in hot embossing. Liu et al. [23] performed MD simulations and found that Van der Waals energy had the greatest effect on interfacial adhesion, and that polymer molecules tended to flow along the sidewalls. In our previous study [24], we used the MD method to elucidate that proper selection of mold insert material and surface modification could effectively reduce interfacial energy and improve the demolding quality. Currently, although MD methods have been widely used to explore molecular motion and to qualitatively analyze molecular morphological evolution at the molecular/atomic level, limited information is available on the interfacial interaction mechanisms that cause structural deformation of polymers in injection molding.

The purpose of this paper is to investigate the internal mechanism of micro-structural demolding deformation of injection molded microfluidic chips. In this study, experiment trials and MD simulations were performed for injection molding of polycarbonate (PC), polymethyl methacrylate (PMMA), cyclic olefin copolymer (COC) and polystyrene (PS), respectively. The morphology and dimensions of the micro-channels and micro-mixing structures were characterized to observe their fabrication quality. In the simulation work, the motion of the polymer molecular chains with different mold insert structures was investigated by analyzing the demolding snapshots. Adhesion work, demolding resistance and potential energy were further investigated to reveal the effect of interfacial interactions on the demolding deformations of polymers. 

## 2. Experiments

### 2.1. Fabrication of Mold Insert

**Figure 1:**
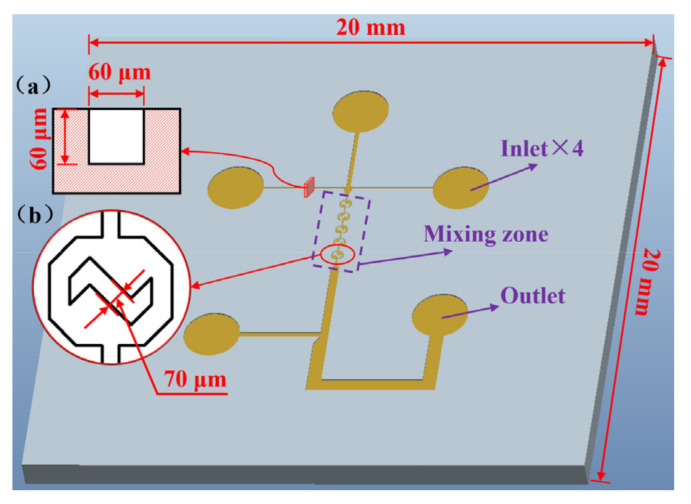
Microfluidic chip designed for heavy metal detection: (**a**) cross section of the micro-channel, (**b**) enlarged view of the micro-mixing structure.

To achieve fast and effective mixing of heavy metal-containing liquids, the structure of a micro-fluidic chip was designed (as shown in Figure 1), including four inlets, one outlet and a mixing zone. The mixing zone is a region containing micro-channels and five liquid micro-mixing areas. The other connecting structures are micro-channels with a depth of 60 μm, a minimum width of 60 μm and a maximum width of 300 μm. The molding quality of the micro-channels and micro-mixing structures of the micro-fluidic chip is the key to achieving heavy metal detection and performance analysis. 

**Figure 2:**
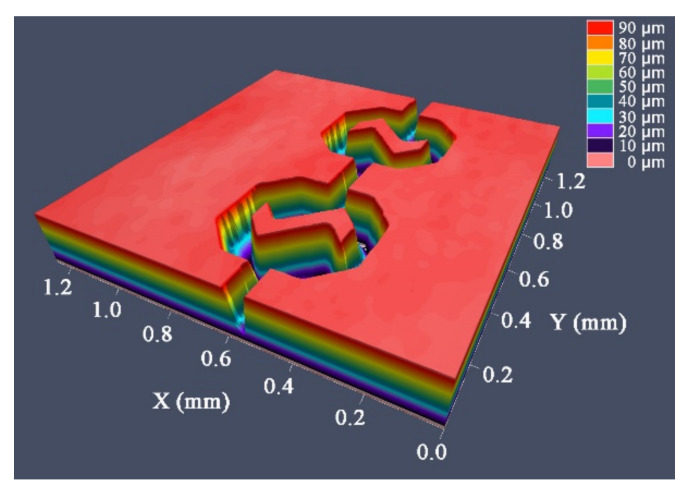
Characterization of the micro-structure of Si master by confocal microscope.

**Figure 3:**
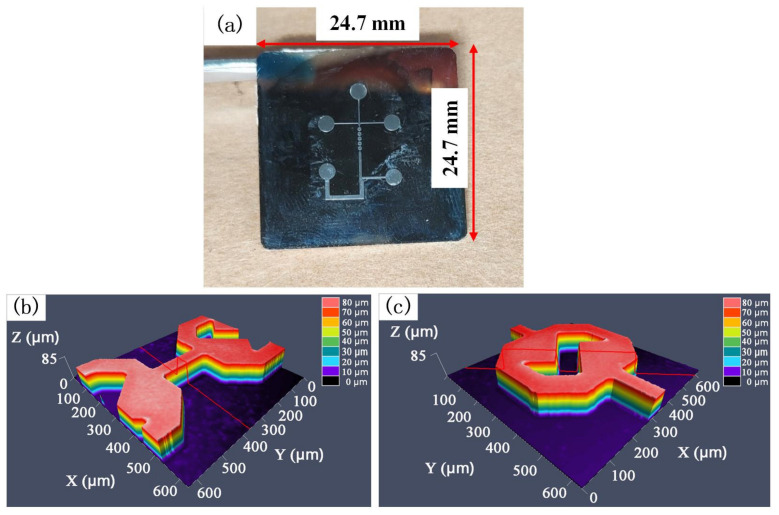
Designed microfluidic chip structure: (**a**) Ni mold insert, (**b**,**c**) micro-channel and micro-mixing structure characterized by confocal microscope, respectively.

Silicon (Si) master was prepared by etching technology. A confocal microscope (Carl Zeiss AG, Oberkochen, BW, GER) was used to characterize the micro-channel and micro-mixing structures of the Si master, as shown in Figure 2. Subsequently, a 50 nm thick conductive layer was deposited on the surface of the Si master by a sputtering coater. Nickel (Ni) was chosen as the material for the mold insert due to its excellent mechanical and chemical properties [25]. Two levels of current density and a self-made electroforming equipment (V-30L, Changsha, China) were used to fabricate the Ni mold insert. First, a small current density of 1 A/dm^2^ was used for 4 h, followed by a current density of 4 A/dm^2^ for 15 h to achieve the desired thickness of 0.8 mm. The Si master was then dissolved with 20 wt% NaOH at 50 °C to obtain the Ni mold insert for injection molding, as shown in Figure 3. The micro-channels and micro-mixing structures were examined with a confocal microscopy to ensure their quality was as required. The average dimensions of the micro-structures of the Si master and Ni mold insert are listed in Table 1.

### 2.2. Injection Molding

The molding experiments of microfluidic chips were conducted using a LD05EH2 (Sodick Co. Ltd., Kanagawa, Japan) injection molding machine. A temperature controller (Yanbang YBM-1H, Dongguan, China) and a water cooling system (Xinyi Electric SIC-3A, Zhongshan, China) were used to control the mold temperature and cooling process in the injection molding, respectively. The mold insert was installed in the fixed mold cavity by a pressing block. Polymers with good transparency and excellent filling properties, such as polycarbonate (PC), polymethyl methacrylate (PMMA), cyclic olefin copolymer (COC) and polystyrene (PS), were selected as the microfluidic chip materials. Table 2 shows the processing parameters based on our previous work [26]. The demolding temperature of the polymers was determined to be 20 °C lower than the glass transition temperature (T_g_) [27]. To ensure the accuracy and repeatability of the experimental results, five samples from each group under the same processing parameters were selected after the twentieth cycle to measure their characteristic dimensions.

### 2.3. Structure Characterization

**Figure 4:**
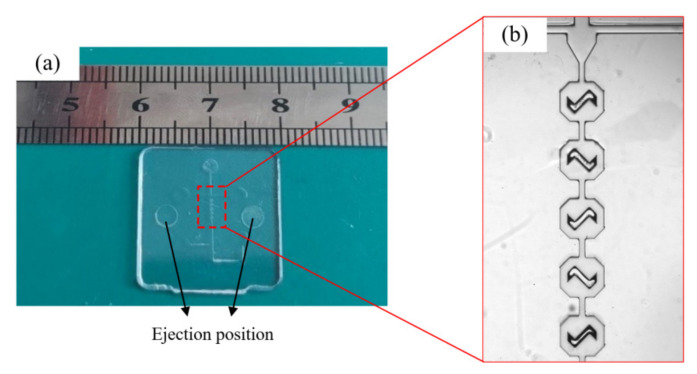
An injection molded product: (**a**) PC microfluidic chip, (**b**) micro-mixing zone observed with light microscopy.

The injection molded PC microfluidic chip (as shown in Figure 4) had a good molding quality with a smooth surface, high transparency and no obvious warpage, fractures or other defects.

**Figure 5:**
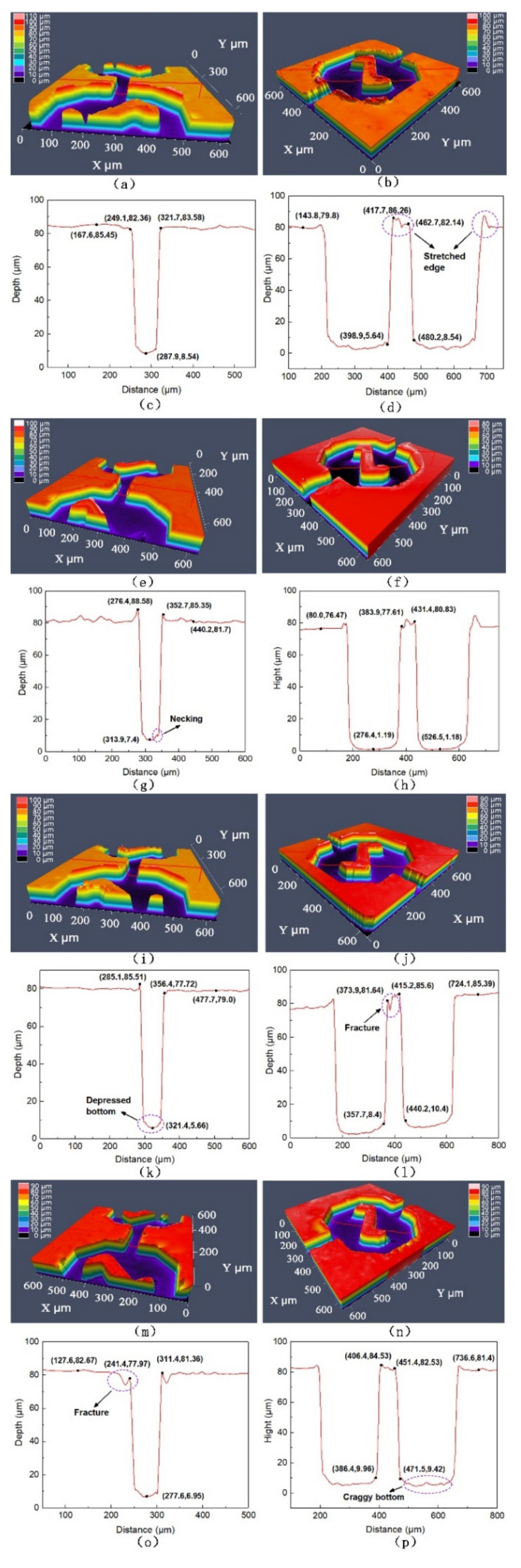
The micro-channels and micro-mixing structures of microfluidic chips of four polymers: (**a**,**b**) PC, (**e**,**f**) PMMA, (**i**,**j**) COC and (**m**,**n**) PS. The cross section profiles of the micro-channels and micro-mixing structures of microfluidic chips of four polymers: (**c**,**d**) PC, (**g**,**h**) PMMA, (**k**,**l**) COC and (**o**,**p**) PS.

The morphology and dimensions of the PC, PMMA, COC and PS micro-structures are shown in Figure 5. Apparently, the micro-channels and micro-mixing structures of microfluidic chips could be well replicated from the Ni mold insert by injection molding. However, the edges of micro-structures of different materials were all elongated after demolding. Compared with other polymers, the PC micro-structures had the most severe elongation deformation, as shown in Figure 5a–d. Due to the low elongation of the materials, the edges of the COC and PS micro-structures were damaged during the demolding process. The tensile deformations and fracture of the edges were mainly attributed to adhesion and friction, which induced demolding resistance [28]. As a result, ridges appeared at the top of the PMMA micro-mixing structure, as shown in Figure 5f,h. It is worth mentioning that the wall perpendicularity of the micro-structures were all well replicated, as seen in the profiles of the molded chips. The surface morphology of the PC and PMMA microfluidic chips presented a wavy shape, while the COC chips had the best flatness. The craggy walls at the bottom of the PC and PS micro-mixing structures would affect the flow of the liquid. There was a slight necking and depressed deformation at the bottom of the micro-structures after demolding [29], which could be relatively neglected. Compared to surface flatness and necking, deformation by stretching and fracture were the molding defects that most affect chip performance. 

In order to quantify the demolding quality, deviation percentages were defined to describe the dimensional differences between the injection molded structure and the mold insert. The calculation formulas were as follows:

(1) $$\delta_{w}=\frac{W_{s}-W_{m}}{W_{m}}\;\times \;100\%$$

where *δ_w_*, *W_s_* and *W_m_* were the width deviation percentage, the average width of the micro-structure and those of the mold insert, respectively.

(2) $$\delta_{h}=\frac{H_{s}-H_{m}}{H_{m}}\;\times \;100\%$$

where *δ_h_*, *H_s_* and *H_m_* were the height deviation percentage, the average height of the micro-structure and those of the mold insert, respectively.

**Figure 6:**
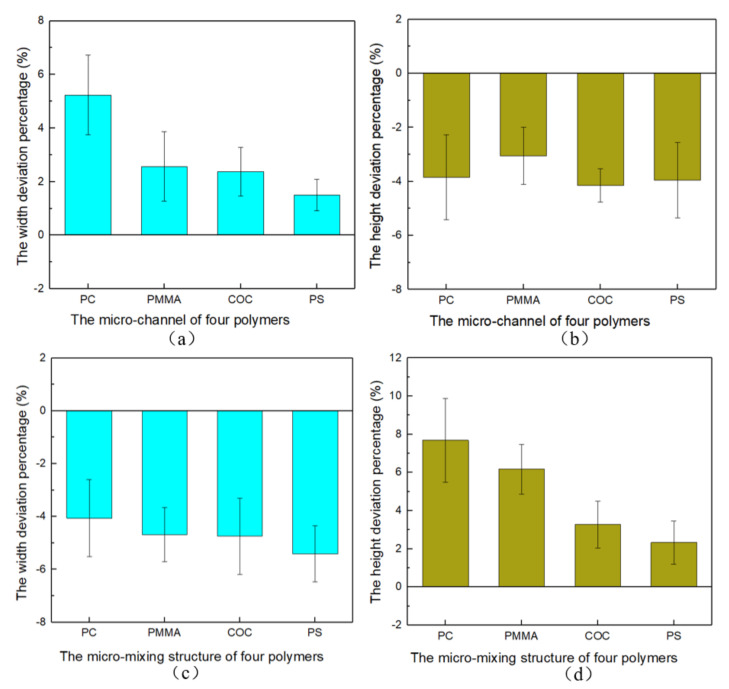
The deviation percentage of PC, PMMA, COC and PS microfluidic chips: (**a**,**b**) micro-channel, (**c**,**d**) micro-mixing structure.

The deviation percentages of the micro-channels and micro-mixing structures compared to the Ni mold insert were calculated, as shown in Figure 6. A positive deviation percentage indicates an increase in the size of the micro-structure after demolding, while the negative value is the opposite. It is clear that the width of the micro-channels increased and the depth decreased, which were determined by the shrinkage of the polymer [30]. Combined with Figure 6, the micro-mixing structures of the four polymers were elongated, which directly reduced the width. The adhesion and friction between the polymer and the mold insert during the demolding process were the main reasons for this result. After demolding, the deviation percentages of the micro-channel height and the width of the micro-mixing structure were relatively stable. However, the deviation percentages of the PC micro-channel width and the micro-mixing structure height were the largest due to the stretched edges, which were 5.24% and 7.68%, respectively. Combining the molding quality and the dimensional fidelity of the micro-structures, the injection molded PC microfluidic chips obtained the worst replication quality. The main reason for the dimensional deviations was the difference in the morphology of the micro-structures, while the intrinsic properties of the polymers were internal factors affecting the reproduction quality. Therefore, the demolding process of different micro-structures and different polymers should be studied separately.

The experimental results showed that deformations such as fractures, stretching and necking occurred after demolding. To effectively explain the internal mechanisms of deformation formation, molecular dynamics (MD) simulations would be used to explore the interfacial interaction from the molecular/atomic scale.

## 3. MD Simulation

### 3.1. Model Construction

A simulation model of injection molding was established, and the interfacial interactions during demolding were analyzed by the MD method. Taking PC as an example, a mono-molecule chain with a polymerization degree of 20 was constructed. An amorphous polymer system with 37 chains and an initial density of 1.20 g/cm^3^ was established. To optimize the energy of the polymer module, the methods of the steepest descent, conjugate gradient and Newton under the NVT ensemble were used, respectively. Then, an annealing and dynamic treatment were performed at 553 K (280 °C) to achieve the melt filling state. The specific parameters for building the PMMA, COC and PS models has been described in detail in our previous work [31].

**Figure 7:**
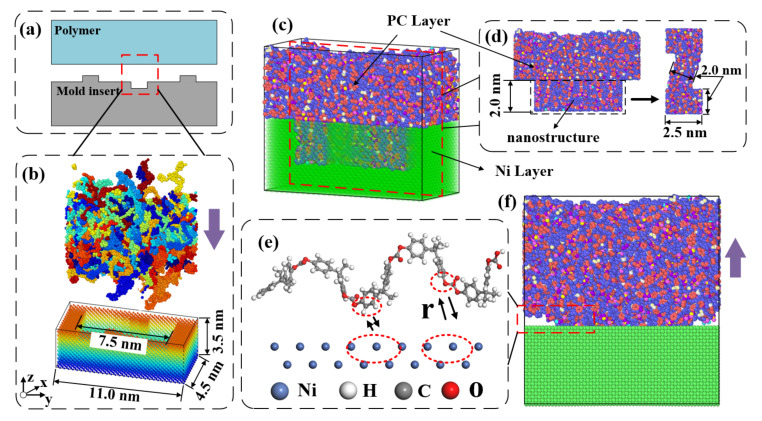
MD model for the injection molding: (**a**) schematic model of filling process, (**b**) initial model of filling process in simulation, (**c**) filling complete model, (**d**) the mixing structure, (**e**) interfacial interaction between the molecular chains and mold insert atoms in demolding, and (**f**) the demolding process.

The Ni mold insert with channel and mixing structures of dimensions 4.5 (x) × 11.0 (y) × 3.5 (z) nm^3^ were obtained by deleting the corresponding atoms from the supercell structure. An initial injection molding model of the mixing structure was constructed, as shown in Figure 7b. Figure 7c showed the fully filled state of the polymer. The interfacial interaction between the polymer molecules and the mold insert atoms is a key factor affecting the demolding quality [24,32], as shown in Figure 7d. In order to be consistent with the real situation, the x-, y- and z-directions were set as periodic boundary, periodic boundary and non-periodic boundary conditions, respectively.

### 3.2. Force Field and Simulation Procedure

The Ni substrate was set as a rigid body using the Cartesian coordinate position before performing the injection molding simulation. The polymer consistent force field (PCFF) was used to describe the bond stretching potential, angular bending potential and dihedral torsion potential between the polymer systems [31]. The non-bonded interfacial interactions were calculated using 12/6 Lennard-Jones (LJ) potential and Coulomb interaction potential energy, as shown in Equation (3):

(3) $$U_{\mathrm{non}-\mathrm{bonded}}=U_{\mathrm{vdw}}+U_{\mathrm{ele}}=4\varepsilon [(\frac{\sigma }{r_{\mathrm{ij}}}{)}^{12}\;(\frac{\sigma }{r_{\mathrm{ij}}}{)}^{6}]+\frac{q_{i}q_{j}}{r_{\mathrm{ij}}}\;(r_{\mathrm{ij}}\;<\;r_{c})$$

where *ε*, *σ*, *r_ij_*, *q*, and *r_c_* were the non-bonded interaction constant, the distance between two atoms in equilibrium, the distance between two atoms at any time, the charge of atoms and the cutoff distance, respectively. 

The adhesion work (*E_work_*) between the polymer and mold insert was used to quantify the interaction strength. The calculation formula was as follows:

(4) $$E_{\mathrm{work}}=\frac{-E_{\mathrm{interaction}}}{A_{c}}=\frac{-[E_{\mathrm{total}}-\left(E_{\mathrm{polymer}}+E_{\mathrm{mold}}\right)]}{A_{c}}$$

where *E_interaction_*, *E_total_*, *E_polymer_*, *E_mold_* and *A_c_* were the interfacial interaction energy, the total energy of the polymer-mold insert system, the surface energy of the polymer, the surface energy of the mold insert and the contact area between the polymer and the mold insert, respectively. 

The simulations were taken in the constant particle number, volume and temperature (NVT) ensemble with a time step of 0.05 fs. The filling time of the molten polymer was determined to be 90,000 fs by the radius gyration of the polymer molecular chains tending to a stable value [33]. The demolding processes were completed within 60,000 fs under the external force of 1.0 kcal/mol·Å (7 × 10^−11^ N) [34]. All the simulations were completed in an atom-based method with an open source software Large-scale Atomic/Molecular Massively Parallel Simulator (LAMMPS 64-bit 9Oct2020-MPI, Sandia National Laboratories, Sandia, Albuquerque, NM, USA).

## 4. Results and Discussion

### 4.1. Demolding Process

**Figure 8:**
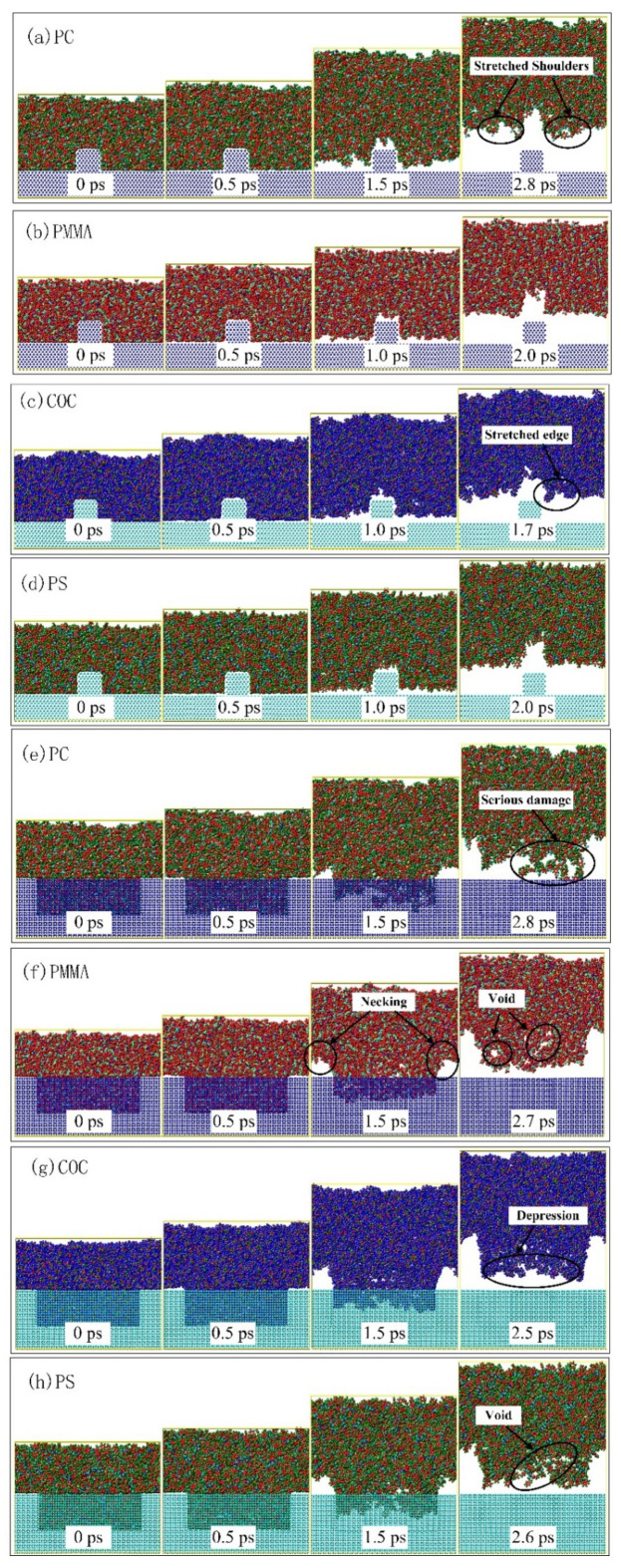
Snapshots of the channels and mixing structures of four polymers: (**a**,**e**) PC, (**b**,**f**) PMMA, (**c**,**g**) COC, (**d**,**h**) PS.

PC, PMMA, COC and PS were completely filled into the channels and mixing structures at 0.0 ps, as shown in Figure 8. Under the action of external force, the polymer layer gradually moved upward in the z-direction until the end of demolding. At about 0.5 ps, the polymer molecules at the shoulders began to separate from the mold insert after overcoming the intermolecular interaction. At the same time, the structures were gradually elongated by the combined effect of adhesion and friction forces. The molecular chains near the shoulders and bottoms were pulled apart and voids appeared, which were obvious during the demolding of the mixing structure. The longest interfacial interaction between the PC molecules and the mold insert resulted in the most severe structural damage after demolding (as shown in Figure 8a,e), which was consistent with the experimental results. The molecular chains near the sidewalls of the mold insert continued to be stretched under the molecular/atomic interactions, although the molecular chains in the horizontal direction were completely separated. The deformation of the mixing structures were more severe compared to the vertical channel structure. The structural design of the mold insert would directly affect the molding quality of the polymer, which was similar to the simulation results of nano-imprint lithography in the literature [35]. It was due to the different contact area between the polymer and the mold insert micro-structure that the interfacial interaction strength and action time of the micro-mixing structure were significantly greater than those of the micro-channel structure. The selection of materials with good molding properties was also not negligible. Obviously, PMMA and PS exhibited a better replication quality after demolding from the snapshots. The simulation results well reflected the micro-structural deformations that appeared in the experiments. 

### 4.2. Adhesion Work

**Figure 9:**
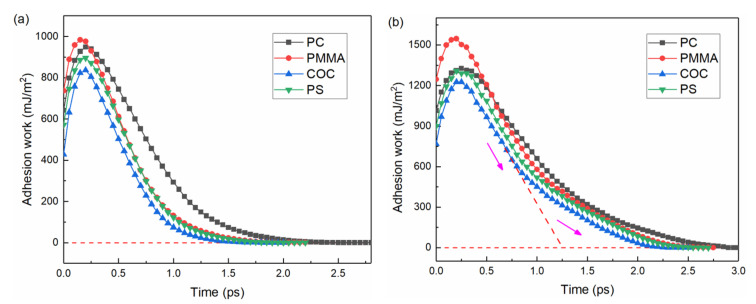
The adhesion work of PC, PMMA, COC and PS in demolding: (**a**) channel structures, (**b**) mixing structures.

The calculated adhesion work between the polymer and the mold insert during the demolding process is shown in Figure 9. The variation trends were basically the same for different polymers and structures. In the initial stage, the adhesion work increased continuously and reached a peak value at about 0.25 ps. At this point, the external force was smaller than the adhesion and friction forces, but larger than the intermolecular forces [24,36]. The polymer structure was stretched and loosened at this stage, although there was no separation between the polymer molecular chains and the mold insert. After 0.25 ps, the distance between the molecular chains and the mold insert gradually increased. The demolding process was completed when the adhesion force dropped to zero, which meant that there was no longer any interfacial interaction between the polymer and the mold insert. Therefore, the demolding process was not a direct separation process, but a gradual separation by overcoming interfacial and intermolecular interactions under the action of external forces. Compared with the channel structure, the total adhesion work of the mixing structure was increased by about 41% to 55%. This brought an increase in the deviation percentage of the micro-mixing structure width after demolding. In addition, the decreasing rate of the adhesion work of the mixing structure tended to decrease in the late stage of demolding. The corresponding decreasing rates of the adhesion work curves for PC, PMMA, COC and PS slowed down around 1.7 ps, 1.0 ps, 0.8 ps and 1.0 ps, respectively, as shown in Figure 9b. At the same time, the action time of the adhesion work increased to some extent. 

### 4.3. Demolding Resistance

**Figure 10:**
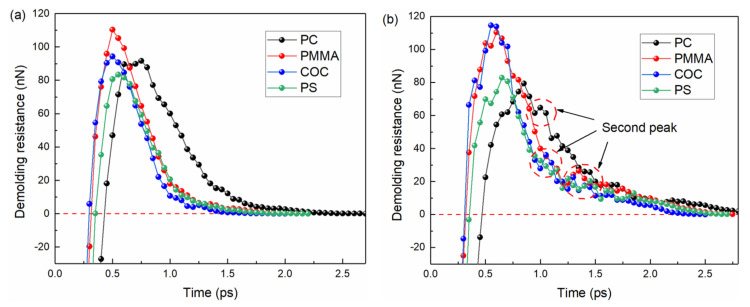
The demolding resistance of PC, PMMA, COC and PS in demolding: (**a**) channel structures, (**b**) mixing structures.

The demolding resistance, consisting of adhesion and friction forces, is the main factor in the deformation of polymer structures [9]. To explore the internal mechanism of molecular chain movement, the demolding resistance was further analyzed. The variations were similar to the adhesion work of the four polymers on different mold insert structures, as shown in Figure 10. Under the combined effect of adhesion and sliding friction, the demolding resistance first gradually increased to a peak value and then decreased to zero as the demolding process proceeded. After the adhesion force reached its peak, the molecular chains started to separate from the mold insert in the horizontal direction, and the sliding friction force started to contribute to the total demolding resistance. In comparison with Figure 9, it could be seen that the effect of friction was significantly greater for COC than for the other three polymers, which lead to a greater demolding resistance than for PC and PS. However, COC had relatively compact molecular chains without severe tensile deformation, as its resistance to demolding was the shortest. The longest action time of the interfacial interaction between the PC and the mold insert produced severe structural incompatibility defects and voids, although the adhesion work was not the greatest. This resulted in the maximum tensile deformation of the PC micro-mixing structure in the experiment, as shown in Figure 6d. The second peak of the demolding resistance of the mixing structure occurred between 1.0 ps and 1.5 ps, which did not occur during the demolding of the channel structure, as shown in Figure 10b. Due to the large contact area, the friction force between the molecular chains and the sidewall of the mold insert was significantly higher during the sliding process. 

### 4.4. Potential Energy

**Figure 11:**
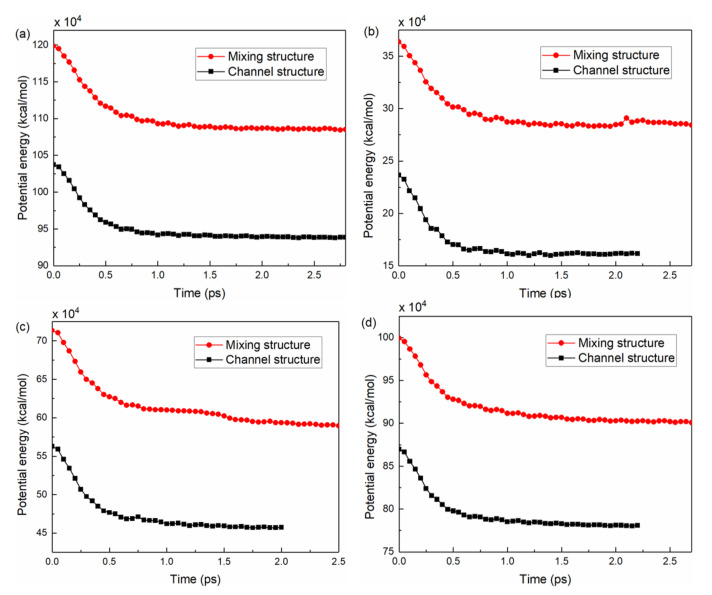
The potential energy of four polymers during the demolding process: (**a**) PC, (**b**) PMMA, (**c**) COC and (**d**) PS.

The potential energies of the four polymers during the demolding stage were calculated, as shown in Figure 11. During the filling stage, the pressure was converted into potential energy and stored in the polymer system. Under the action of external forces, the potential energy was released and decreased rapidly until 0.5 ps. Accordingly, the polymer structure changed from a compact state to a loose state. The potential energy trends of the four polymers on the mold inserts with different structures were essentially similar. It could be noted that the potential energy of the mixing structure before and after demolding were significantly larger than that of the channel structure. The potential energies of PC and PS were higher compared to the other two polymers. This may be due to the relatively high structural stiffness, because of the presence of polar groups such as benzene rings in the molecular chains [34]. The main cause of polymer deformation could not be attributed exclusively to the work of adhesion; the release of potential energy was also an important aspect. 

The changes in potential energy were calculated to quantitatively characterize the deformations of the polymer molecular chains. Table 3 demonstrated the potential energy P_b_ and P_a_ of the four polymers before and after demolding, respectively. The potential energy of PMMA was the smallest, which indicated that this polymer system had the lowest cohesive energy [37]. The PMMA channel and mixing structures with the smallest change in potential energy before and after demolding had the smallest deformation, as shown in the snapshots in Figure 8b,f. In general, the change in potential energy before and after demolding was greater for the mixing structure of the four polymers than for the channel structure; the serious stretching deformation of the micro-mixing structure in the experiment was further explained. 

## 5. Conclusions

In this study, demolding experiments of the injection molded microfluidic chips for heavy metal detection were conducted, and the internal mechanisms of polymer demolding deformation were analyzed by MD simulations. The main conclusions are as follows:

(1) Some defects such as stretching and fracture occurred at the micro-structure edges of the injection-molded microfluidic chips. Due to the longest action time of adhesion work and demolding resistance, the PC micro-structures had the worst replication quality with the deviation percentages of 5.24% and 7.68% for the micro-channel width and micro-mixing structure height, respectively. Compared with the micro-channels, the molding quality of the mixing structure is more difficult to guarantee. 

(2) The MD simulations showed that the polymer molecular chains close to the mold insert atoms elongated continuously under the combined effect of external forces and demolding resistance. Due to the high adhesion work and friction force caused by the large contact area, an increase in the deviation percentage of the micro-mixing structure width occurred after demolding. The demolding deformations of the microfluidic chip micro-structures of the four polymers in the experiment were effectively explained. 

(3) The main cause of polymer deformation could not be attributed exclusively to the adhesion work and friction force; the potential energy was also an important aspect. The PMMA structure has the least change in potential energy, although the PMMA has the greatest adhesion work. The change of potential energy of the mixing structure apparently increased compared to the channel structure, which further explained the serious stretching deformation of the micro-mixing structure in the experiment. 

The internal mechanism that causes the polymer deformation is explained in this study, which would provide theoretical guidance for high-quality injection molding of polymer micro-structures. In future work, studies will be carried out to reduce the interfacial interactions between the polymer and the mold insert, aiming to improve the quality of injection molded microfluidic chips. 

## Figures and Tables

**Table 1:** The average dimensions of Si master and Ni mold insert.

Type	Si Master (μm)		Ni Mold Insert (μm)	
	Average Width	Average Depth	Average Width	Average Depth
Micro-channel	58.01	76.07	60.16	77.56
Micro-mixing structure	73.10	76.46	70.14	78.04

**Table 2:** Processing parameters of the injection molding experiments.

Polymer	Melt Temperature (°C)	Mold Temperature (°C)	Injection Rate (cm^3^/s)	Packing Pressure (Mpa)	Demolding Temperature (°C)
PC	280	155	32	110	130
PMMA	260	115	30	110	90
COC	270	140	28	110	115
PS	240	105	30	110	80

**Table 3:** Changes in the potential energy of four polymers on different structural mold inserts.

Type	Polymer	P_b_ (×10^4^ kcal/mol)	P_a_ (×10^4^ kcal/mol)	Change in the Potential Energy (×10^4^ kcal/mol)
Channel structure	PC	103.8	93.87	9.88
	PMMA	23.67	16.18	7.49
	COC	56.27	45.73	10.54
	PS	86.98	78.08	8.9
Mixing structure	PC	119.9	108.5	11.34
	PMMA	36.36	28.42	7.94
	COC	71.36	58.95	12.41
	PS	99.94	90.08	9.86

## Data Availability

Not applicable.
